# Composition and Anti-*Helicobacter pylori* Properties of Essential Oils Obtained from Selected *Mentha* Cultivars

**DOI:** 10.3390/molecules28155690

**Published:** 2023-07-27

**Authors:** Bartłomiej Piasecki, Izabela Korona-Głowniak, Anna Kiełtyka-Dadasiewicz, Agnieszka Ludwiczuk

**Affiliations:** 1 Department of Pharmacognosy with the Medicinal Plant Garden, Medical University of Lublin, 1 Chodźki Str., 20-093 Lublin, Poland; bartlomiejpiasecki@gmail.com; 2 Department of Pharmaceutical Microbiology, Medical University of Lublin, 1 Chodźki Str., 20-093 Lublin, Poland; iza.glowniak@umlub.pl; 3 Department of Plant Production Technology and Commodity, University of Life Sciences in Lublin, 20-950 Lublin, Poland; anna.kieltyka-dadasiewicz@up.lublin.pl

**Keywords:** mint chemotypes, menthol, carvone, *Helicobacter pylori*

## Abstract

*Helicobacter pylori* infections are highly common amongst the global population. Such infections have been shown to be the cause of gastric ulcers and stomach carcinoma and, unfortunately, most cases are asymptomatic. Standard treatment requires antibiotics such as metronidazole or azithromycin to which many strains are now resistant. *Mentha* species have been used as a natural treatment for gastrointestinal diseases throughout history and essential oils (EOs) derived from these plants show promising results as potential antimicrobial agents. In this study, EOs obtained from the leaves and flowers of five cultivars of *Mentha* × *piperita* and *M. spicata* were examined by GC-MS. The investigated mints are representatives of four chemotypes: the menthol chemotype (*M.* × *piperita* ‘Multimentha’ and *M.* × *piperita* ‘Swiss’), the piperitenone oxide chemotype (*M.* × *piperita* ‘Almira’), the linalool chemotype (*M.* × *piperita* ‘Granada’), and the carvone chemotype (*M. spicata* ‘Moroccan’). The chemical composition of EOs from mint flowers and leaves was comparable with the exception of the Swiss cultivar. Menthol was the most abundant component in the leaves while menthone was highest in flowers. The *H. pylori* ATCC 43504 reference strain and 10 other *H. pylori* clinical strains were examined for their sensitivity to the EOs in addition to their major monoterpenoid components (menthol, menthone, carvone, dihydrocarvone, linalool, 1,8-cineole, and limonene). All tested mint EOs showed inhibitory activity against both the reference *H. pylori* ATCC 43504 strain (MIC 15.6–31.3 mg/L) and clinical *H. pylori* strains (MIC_50/90_ 31.3–250 mg/L/62.5–500 mg/L). Among the reference monoterpenes, menthol (MIC_50/90_ 7.8/31.3 mg/L) and carvone (MIC_50/90_ 31.3/62.5 mg/L) had the highest anti-*H. pylori* activity, which also correlated with a higher activity of EOs containing these compounds (*M.* × *piperita* ‘Swiss’ and *M. spicata* ‘Moroccan’). A synergistic and additive interaction between the most active EOs/compounds and antibiotics possibly points to a new plant-based anti-*H. pylori* treatment.

## 1. Introduction

*Helicobacter pylori* is a Gram-negative bacillus of helical shape and length of approximately 3 µm. It is responsible for many gastric diseases including ulcers, adenocarcinoma, and mucosa-associated lymphoid tissue lymphoma. These diseases are obviously infectious and occur as a consequence of direct human to human transmission [1]. By producing the urease enzyme, *H. pylori* is capable of increasing the pH value in its surrounding harsh gastric environment and surviving in it. This bacillus uses flagella to translocate through the stomach mucus and it can create a biofilm, both of which result in problematic eradication. Eradication of *H. pylori* is not necessary when there are no symptoms even if the patient is diagnosed as *H. pylori* positive. Complete removal of *H. pylori* from the stomach mucus requires the use of multiple antibiotics (clarithromycin, amoxicillin, metronidazole, levofloxacin) and drugs that increase stomach pH (for example, bismuth subsalicylate and proton-pump inhibitors) [2]. Although some combinations are effective against both antibiotic-resistant and non-resistant strains, resistance of *H. pylori* is becoming a considerable problem [3]. Eradication is an important tool to prevent gastric carcinoma and to improve the quality of life of patients with ulcers and hyperacidity, therefore it requires new methods to be effective in the future.

The Maastricht V/Florence Consensus report [1] does not mention any plant-derived medicament in anti-*H. pylori* treatment, although it clearly points out that standard antibiotic/chemotherapeutic-based therapies are becoming less effective. Much research has shown that phytopharmaceuticals have value in the search for safe and active compounds not only in *H. pylori* infections but also in bactericidal infections in general. The studies of Mahady et al. [4] and Bhamarapravati et al. [5] showed that many plant extracts are effective in growth inhibition of *H. pylori* and that they may have some chemoprotective properties resulting in a reduced incidence of gastric cancer. Much research has also been conducted exploring essential oils (EOs) that show significant inhibition effects on *H. pylori* activity and exhibiting gastroprotective properties [6,7,8,9,10]. Studies have demonstrated that EOs increase the production of mucus in the stomach and duodenum, which prevents the development of ulcers [9,10,11]. Additionally, research conducted by Ohno and coworkers [12] showed that EOs are bactericidal against *H. pylori* without the development of acquired resistance. This finding suggests that EOs may be prime candidates as agents to treat *H. pylori* infection.

The genus *Mentha* is a member of the Lamiaceae family and its species are widespread aromatic herbs. The number of these species within *Mentha* is uncertain due to a variety of different classification schemes, which range from 13 to 18 representatives, and this genus still requires revision [13]. Some representatives of *Mentha* are common (*Mentha* × *piperita*, *M. arvensis*, *M. spicata*, *M. longifolia*) while others are endemic (*M. gattefossei* and *M. requienii*). Hybrids frequently occur and many create new subspecies [14]. A high concentration of volatile compounds and polyphenols make them remedies for such diseases as stomachache, indigestion, flatulence, nausea, and urinary and digestive tract infections. It is also possible to use EOs distilled from mints for inhalation in the treatment of upper respiratory tract infections. There is a paucity of data on the anti-*H. pylori* activity of mint EOs, despite the fact that these oils are amongst the most popular. Research conducted by Imai et al. [15] showed that EOs from peppermint and spearmint are able to inhibit the growth and viability of *H. pylori* in vitro. As shown in our previous work, commercial *M.* × *piperita* EO inhibited the growth of *H. pylori* with an MIC value of 62.5 mg/mL [16]. Considering the fact that the chemical composition of mints and their EOs is highly diverse, and that the existence of at least four chemotypes has been confirmed [17], comparing the anti-*H. pylori* activity and correlating it with the chemical composition will considerably improve our knowledge of the mint group.

For the present studies, representatives of four different mint chemotypes were chosen and, in addition, the EOs were obtained from the leaves and flowers separately. We addressed the following questions: (1) Is the chemical composition of the EOs obtained from mint leaves and flowers the same? (2) Are mint EOs active against reference and clinical strains of *H. pylori*? If so, which of the recognized mint chemotypes show the highest effectiveness as antimicrobial agents against *H. pylori*?

## 2. Results

### 2.1. GC-MS Results of Mint Essential Oils

Nine samples of mint grown in Poland were hydro-distilled to obtain EO. A list of the investigated mints with EO yields is shown in Table 1. GC-MS analysis of all obtained EOs was performed, and identified volatile components are listed in Table 2 in order of their elution from a ZB-5MS column.

The data showed considerable variability in EO content and chemical composition between the mints. The content of EOs ranged from 1.19% for leaves of *Mentha* × *piperita* ‘Almira’ (MpAlL) to 3.77% for flowers of *M.* × *piperita* ‘Granada’. Mint flowers are richer in EOs. As shown in Table 2, in the EO obtained from MpAlL, a representative of the piperitenone oxide chemotype, 16 compounds were identified. The two most characteristic components of this EO were piperitenone and piperitenone oxide. The relative percentage of these compounds, among all components detected, was 76.5%. Among volatiles identified in EOs from leaves and flowers of *M.* × *piperita* ‘Granada’ (MpGrL and MpGrF), the most characteristic were linalool and its acetate. The relative percentages of both compounds in these EOs were 65.4% (leaves) and 71.5% (flowers). Noteworthy are two further pairs of monoterpene alcohols and their acetates, namely geraniol and geranyl acetate and nerol and neryl acetate. These components were identified only in this peppermint cultivar.

Two representatives of the menthol chemotype are the subject of the present studies, namely *M.* × *piperita* ‘Swiss’ (MpSwL and MpSwF) and *M.* × *piperita* ‘Multimentha’ (MpMmL and MpMmF). GC-MS analysis of EOs hydrodistilled from leaves and flowers of both cultivars showed that menthol and menthone are the most abundant components. The percentages of both compounds in these four EOs ranged from 56% for MpSwL and 58% for MpMmF to 71% for MpSwF and MpMmL. It is noteworthy that *M.* × *piperita* ‘Swiss’ was the only specimen that showed differences in the ratio of the main compounds of EOs between leaves and flowers. For leaves, menthol and menthone with concentrations of 45.2% and 10.8%, respectively, were the main compounds, while for flowers the main monoterpenes were menthone (48.3%) and menthol (23.2%). Menthone was the major constituent of EOs from the ‘Multimentha’ cultivar.

The last two EOs were obtained from the leaves and flowers of another mint species, *M. spicata*. The cultivar ‘Moroccan’ was selected for research studies. In both essential oils, MsMoL and MsMoF, carvone, and (*Z*)-dihydrocarvone, together with limonene, were the major volatile constituents. The relative amounts of these components in both EOs ranged from 77% for leaves to 80% for flowers.

All analyzed EOs showed a large predominance of oxygenated monoterpenoids (alcohols, ketones, and esters). As shown in Table 2, in six of the nine EOs, monoterpene ketones were the most abundant components. Monoterpene alcohols together with esters are characteristic for peppermint belonging to the linalool chemotype but also for leaves of *M.* × *piperita* ‘Swiss’. In addition to a high concentration of menthol, almost 12% menthyl acetate was detected in this EO.

### 2.2. Antimicrobial Activity of Mint Essential Oils and Reference Compounds

As shown in Table 3, all tested mint EOs showed inhibitory activity against both the reference *H. pylori* ATCC 43504 strain (minimum inhibitory concentration (MIC) 15.6–31.3 mg/L) and clinical *H. pylori* strains (minimum concentration at which 50% and 90% of the isolates were inhibited (MIC_50_/_90_) 31.3–250 mg/L/62.5–500 mg/L). For all investigated EOs, the activity against the reference strain was considerably higher than against the clinical strains.

The highest activity against the reference strains was detected for EOs obtained from *M.* × *piperita* cultivars ‘Almira’ (MpAlL) and ‘Swiss’ (MpSwL and MpSwF), as well as from flowers of *M. spicata* ‘Moroccan’ (MsMoF). For the clinical isolates, the activity of both EOs from *M. spicata* ‘Moroccan’ was one of the strongest measured and can be compared only with EOs obtained from *M.* × *piperita* ‘Swiss’. As mentioned in a previous subsection, *M.* × *piperita* ‘Swiss’ was the only specimen that showed differences in the ratio of the main compounds of EOs between the leaves and flowers. For the leaves, menthol was the main compound, while for flowers it was menthone. Although it seemingly did not affect the activity of both *M.* × *piperita* ‘Swiss’ EOs against the reference ATCC 43504 strain, the differences in activity against the clinical strains are in favor of EO obtained from leaves, containing the highest amount of menthol. *M.* × *piperita* ‘Multimentha’ EO from the leaves (MpMmL) had higher activity than the sample from flowers (MpMmF), despite the concentrations of menthone being nearly the same: 48.9% in leaves and 49.3% in flowers (see Table 2).

The *H. pylori* ATCC 43504 reference strain and 10 other *H. pylori* clinical strains were also examined for their sensitivity to the major components present in EOs, specifically menthol, menthone, linalool, carvone, dihydrocarvone, 1,8-cineole, and limonene. Among the reference compounds, menthol (MIC_50_/_90_ 7.8/31.3 mg/L) and carvone (MIC_50_/_90_ 31.3/62.5 mg/L) had the highest anti-*H. pylori* activity, which was also reflected by the higher activity of EOs containing these compounds: *M.* × *piperita* ‘Swiss’ and *M. spicata* ‘Moroccan’, respectively (Table 3).

**Figure 1:**
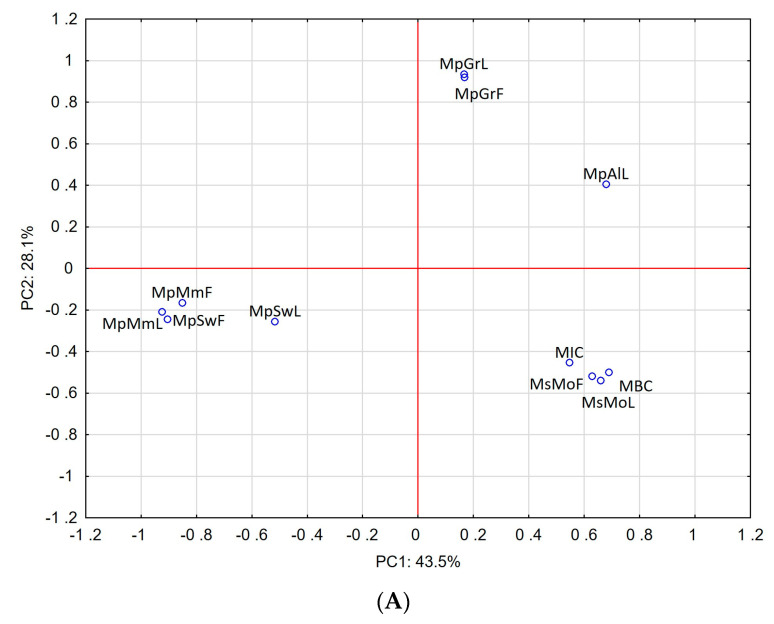

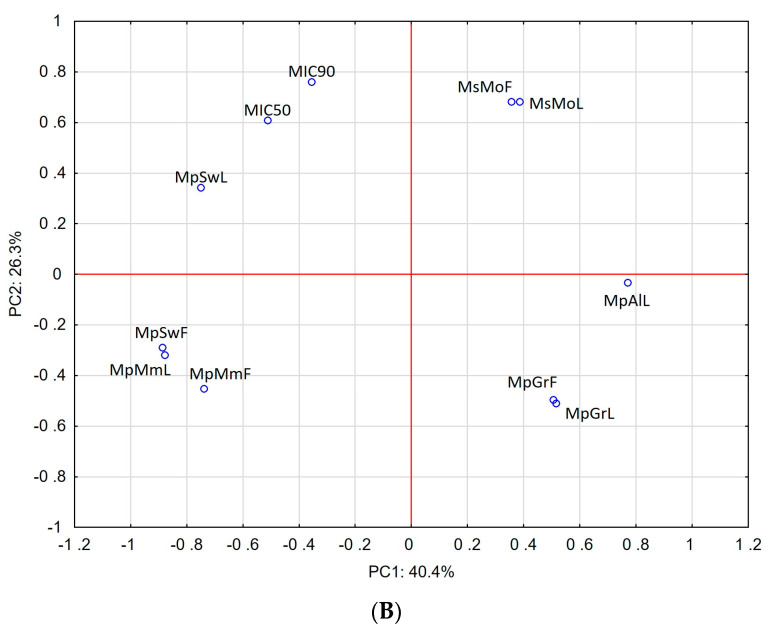
Principal component analysis of mint EOs based on their chemical composition and anti-*H. pylori* activity. (**A**) Correlation between EO composition and anti-*H. pylori* activity of reference strain, (**B**) correlation between EO composition and anti-*H. pylori* activity of clinical strains.

Our results were further supported by principal component analysis (PCA), which was applied to compare the anti-*H. pylori* activity of the investigated EOs with their chemical composition. The results are presented in Figure 1. The scores plot (non-normalized, without rotation) observed for the first two PCs, presented in Figure 1A, showed a good correlation of highest microbial activity against the reference strain (ATCC 43504) and the carvone-rich EOs MsMoL and MsMoF. For the clinical isolates, the best correlation was also observed for carvone-rich EOs but especially for the menthol-rich EO, which was obtained from the leaves of *M.* × *piperita* ‘Swiss’ (Figure 1B).

To estimate the general combination effects between the most active EOs/compounds and antibiotics, such as metronidazole and clarithromycin, on the antibacterial activity against clinical isolates, the fractional inhibitory concentration (FIC) was determined by a checkerboard study and fractional inhibitory concentration indices (FICIs) were calculated. FICI values were interpreted according to EUCAST [18] as a synergistic effect when FICI ≤ 0.5; an additive effect when 0.5 < FICI < 1; an indifferent effect when 1 < FICI < 4; and an antagonistic effect when FICI > 4. The results are shown in Table 4.

The results showed additive or synergistic effects between EOs and the antibiotics. According to the EUCAST interpretation, the interaction between essential oils from *M. spicata* ‘Moroccan’ flowers and *M.* × *piperita* ‘Swiss’ leaves and metronidazole revealed synergistic or additive effects against reference and clinical *H. pylori* strains with FICI values of 0.28–0.625. The combination of menthol or (*R*)-(−)-carvone and metronidazole showed synergistic effects (FICI values 0.094–0.31) against *H. pylori* strains. The combination of both EOs as well as menthol and (*R*)-(−)-carvone with clarithromycin resulted in additive antibacterial activity against *H. pylori*, with FICI values of 0.51–1.00.

## 3. Discussion

Due to the increasing number of antibiotic-resistant *H. pylori* strains, the search for safe and effective agents is necessary. EOs seem to be good candidates, especially since research has shown that *H. pylori* does not become resistant to such mixtures of volatile compounds obtained by distillation of various plants. Research conducted by some Japanese scientists has shown that resistance to lemongrass (*Cymbopogon citratus*) EO did not develop even after ten sequential passages, whereas resistance to clarithromycin developed under the same conditions [12]. The lemongrass EO was the most active against *H. pylori* strains among thirteen commercial EOs tested in vitro. Examination of the bactericidal effect of this EO in vivo also showed that one of the ten mice in the group that received lemongrass oil was completely cured [12]. In the in vivo experiment performed by Harmati et al. [6], the 2:1 mixture of *Satureja hortensis* and *Origanum vulgare* subsp. *hirtum* EOs successfully eradicated *H. pylori* in 70% of the mice. Another study by Bergonzelli et al. [19] showed anti-*H. pylori* potential of 30 among 60 analyzed EOs. They identified 15 EOs with strong anti-*Helicobacter* activities with MBCs ranging from 20 to 100 mg/L after 24 h of incubation and established that the bactericidal activities are enhanced at acidic pH. Among the most active EOs were lemongrass, oregano, and thyme [19]. Although scarce, there are also some data concerning mint EOs. Weseler et al. [20] showed that peppermint EO was active against *H. pylori* with an MIC value of 135.6 mg/L and an MBC of 542.2 mg/L. Reichling et al. [21] indicated that *H. pylori* was sensitive to the EO from *M. spicata* with MIC values ranging from 50–100 mg/L, whilst an *M. arvensis* EO had an MIC of 100 mg/L. Other studies have shown that commercial peppermint EO inhibited the growth of *H. pylori* at an MIC value of 62.5 mg/L [16].

These in vitro and in vivo data indicate that EOs show efficiency against *H. pylori* infections, mainly oils derived from plants belonging to the Lamiaceae, like thyme, oregano, savory, and mint. EOs from plants classified in this family also predominate in the few available review articles on EOs active against *H. pylori* [22,23,24,25]. However, Lamiaceae plants are known for their chemical polymorphism, and therefore the knowledge of the chemotype of an EO is important [26].

To the authors’ best knowledge, there are no studies linking anti-*H. pylori* activity with chemotypes of Lamiaceae plants. Based on all the mentioned facts, we chose mint for our research, as the least studied plant from the Lamiaceae family, and asked the question: Which of the recognized mint chemotypes has the strongest effect on reference and clinical *H. pylori* strains?

Our data showed that, according to O’Donnell et al. [27], investigated mint EOs showed moderate to strong activity against both the reference and clinical *H. pylori* strains. The activity was dependent on the mint chemotype. Among the menthol, linalool, piperitenone oxide, and carvone chemotypes, only the menthol- and carvone-rich EOs had strong and good anti-*H. pylori* activity. EOs from mints classified in the linalool and piperitenone oxide chemotypes showed moderate activity against *H. pylori*. Our previous study [17] indicated that EOs obtained from mints belonging to the piperitenone oxide chemotype exhibited significant bacteriostatic activity against *Staphylococcus epidermidis*, the most frequent cause of infections in medical devices. Chemotype-dependent activity against methicillin-resistant *Staphylococcus aureus* of EOs from different *Cymbopogon* species was also observed. The most active EO was from *C. flexuosus*, classified in the citral chemotype [28]. EOs are characterized by a large variety of compounds found in them. One EO may contain a dozen or even several dozen compounds with different concentrations and properties. Most of the EOs contain the dominant constituent but the antimicrobial activity of a particular EO does not always depend on the activity of the major component. Sometimes the activity is the result of the antimicrobial properties of all EO constituents [29]. In the case of mint EOs, the most characteristic components occurring in all of the recognized chemotypes are oxygenated monoterpenoids, and among them alcohols, ketones, esters, and oxides were detected. Of the major components present in EOs, menthol, menthone, linalool, carvone, dihydrocarvone, 1,8-cineole, and limonene, menthol and carvone had the highest activity which was similar to that of the antibiotic metronidazole. Menthol and carvone activity was also reflected by a higher activity of EOs containing these compounds in *M.* × *piperita* ‘Swiss’ and *M. spicata* ‘Moroccan’, respectively. This finding is in agreement with the highest antimicrobial activity of carvacrol, thymol, and citral, the major components of the EOs from *Origanum*, *Thymus*, and *Cymbopogon* species, respectively [19]. Al-Sayed and coworkers showed that the presence of dominant compounds in *Piper* EOs may also explain their anti-*Helicobacter* activity [30].

The existence of synergism in the antimicrobial activity of the most active compounds/EOs and the antibiotics clarithromycin and metronidazole was determined against clinical isolates of *H. pylori*. The results showed additive or synergistic effects. The synergistic effect of marjoram and petitgrain mandarin oils on anti-*H. pylori* activity was observed by Elkousy and colleagues [31]. The combined oil sample showed the highest inhibitory effect against *H. pylori*. Clarithromycin demonstrated the same MIC value as the combined oil, at the same concentration used [31].

Terpenoids, which are the main constituents of EOs, are weakly (hydrocarbons) to moderately (alcohols, ketones) soluble in water, but they are dissolvable in the phospholipid membrane. The antimicrobial mechanism of EOs is not fully understood, but it could be explained by their ability to disrupt or penetrate lipid structure, causing a loss of membrane integrity [32,33]. Our research showed that menthol was the most active ingredient in peppermint oils. This compound, together with thymol, was investigated for the mechanism of action against *Escherichia coli* (Gram-negative) and *Staphylococcus aureus* (Gram-positive) by Trombetta et al. [32]. These authors concluded that activity of monoterpenes depended on their water solubility and the type of cell wall they interacted with. It was suggested that it is probable that some of the volatiles can penetrate into bacteria and cause further cellular damage [32].

Published data suggest that EOs are moderately safe and easy to use as phytopharmaceuticals with bactericidal properties that have a low risk of resistance development [33,34,35,36,37]. However, they require correct dosing and formulation depending on the route of administration and the type of infection, for example, microencapsulation or suspension for oral administration and emulsification for a dermal application [38,39]. Numerous reports indicate that different EOs are investigated yearly as hits and potential leads against pathogenic bacteria.

## 4. Materials and Methods

### 4.1. Plant Material and Essential Oils

Essential oils were obtained from 4 different cultivars of *Mentha* × *piperita*: ‘Multimentha’, ‘Swiss’, ‘Almira’, and ‘Granada’, and additionally one cultivar of *M. spicata* named ‘Moroccan’. Mints were cultivated in the garden of the Research and Science Innovation Center in Wola Zadybska near Lublin (Poland) (51°44′49″ N 21°50′38″ E) and collected in 2017 from June to August. After drying, plants were divided into leaves and flowers except for *M.* × *piperita* ‘Almira’, for which only leaves were obtained. Due to the variety of morphological features, which are characterized by very small leaves and flowers, it is difficult and sometimes impossible to separate the flowers. Voucher specimens were deposited in the Research and Science Innovation Center. The essential oils of air-dried plant materials were obtained by hydrodistillation for 2 h in a Deryng-type apparatus. The oils were stored in tightly sealed 1.5 mL amber vials at 4 °C prior to analyses.

### 4.2. GC-MS Analysis

Chromatographic analysis was performed with a Shimadzu GC-2010 Plus instrument coupled to a Shimadzu QP2010 Ultra mass spectrometer. Compounds were separated on a ZB-5 MS fused-silica capillary column (30 m, 0.25 mm i.d.) with a film thickness of 0.25 mm (Phenomenex). The following oven temperature program was initiated at 50 °C, held for 3 min, then increased at the rate of 8–250 °C/min, and held for 2 min. The spectrometer was operated in EI mode; the scan range was 40–500 amu, the ionization energy was 70 eV, and the scan rate was 0.20 s per scan. The injector, interface, and ion source were kept at 250, 250, and 220 °C, respectively. Split injection was conducted with a split ratio of 1:20 and helium was used as the carrier gas at a 1.0 mL/min flow rate. The retention indices were determined in relation to a homologous series of *n*-alkanes (C_8_–C_24_) under the same operating conditions. Compounds were identified using a computer-supported spectral library (MassFinder, NIST 2011), mass spectra of reference compounds, as well as MS data from the literature [40,41]. Compound identities were confirmed by comparison of retention indices with reference compounds and published data [41].

### 4.3. Microbiological Study

The obtained EOs and their components (menthol, menthone, carvone, dihydrocarvone, linalool, 1,8-cineole, and limonene) were tested for antibacterial activity against *H. pylori* ATCC 43504 as well as 10 other *H. pylori* clinical strains. The MIC and MBC values of EOs were determined using the two-fold microdilution method at a concentration range from 3.9 to 1000 mg/L. Resazurin 0.01% was added after incubation to reflect the growth of bacteria as described elsewhere [16]. MIC50 and MIC90, which represent the concentrations shown to be effective for ≥50% and ≥90% of isolates tested, respectively, were determined. In this study, no bioactivity was defined as an MIC > 1000 µg/mL, mild bioactivity as an MIC in the range 501–1000 µg/mL, moderate bioactivity with MIC from 126 to 500 µg/mL, good bioactivity as an MIC in the range 26–125 µg/mL, strong bioactivity with MIC between 10 and 25 µg/mL, and very strong bioactivity as an MIC < 10 µg/mL [27].

To estimate the general combination effect between EOs/compounds and other antibiotics, such as metronidazole and clarithromycin, on the antibacterial activity against clinical isolates, the fractional inhibitory concentration (FIC) was determined by a checkerboard study [42]. The experiments were repeated in triplicate. Representative data are shown.

### 4.4. Statistical Analysis

All analyses were performed in triplicate in order to prove their reproducibility. To correlate the anti-*H. pylori* activity of the investigated EOs with their chemical composition, principal component analysis (PCA) was performed using STATISTICA 13 (StatSoft, Cracow, Poland). The analysis was carried out separately for the reference strain and for the clinical strains.

## 5. Conclusions

In conclusion, mint EOs, especially these obtained from *M. spicata* ‘Moroccan’ and *M.* × *piperita* ‘Swiss’, have good to strong bactericidal effects on the growth of *H. pylori*. Very strong anti-*H. pylori* activity against the clinical strains was shown for menthol, the major component of EO from the leaves of *M.* × *piperita* ‘Swiss’. This activity was the same as for the metronidazole used in the study. The monoterpene ketone carvone was second only to menthol in showing the highest antimicrobial activity. Additionally, synergistic and additive interactions between the most active EOs/their compounds and antibiotics were observed.

The results indicate the value of further research and development of new plant-based anti-*H. pylori* treatments. These results indicated that mint EOs may be a valuable therapeutic agent acting supportively against *H. pylori* infections and reducing the cost of treatment of such infections.

## Figures and Tables

**Table 1:** List of the investigated mints with abbreviations and EO yields.

No.	Plant Name, Organ	Code	EO Yield [%]
1	*M.* × *piperita* ‘Granada’, leaves	MpGrL	2.23
2	*M.* × *piperita* ‘Granada’, flowers	MpGrF	3.77
3	*M.* × *piperita* ‘Multimentha’, leaves	MpMmL	2.26
4	*M.* × *piperita* ‘Multimentha’, flowers	MpMmF	3.75
5	*M.* × *piperita* ‘Swiss’, leaves	MpSwL	2.16
6	*M.* × *piperita* ‘Swiss’, flowers	MpSwF	3.34
7	*M.* × *piperita* ‘Almira’, leaves	MpAlL	1.19
8	*M. spicata* ‘Moroccan’, leaves	MsMoL	2.20
9	*M. spicata* ‘Moroccan’, flowers	MsMoF	2.64

**Table 2:** Chemical composition of the essential oils obtained from the examined mint cultivars. RI_exp_.—retention index on ZB-5MS column, RI_lit_.—retention index from the literature (MassFinder, NIST). For abbreviations of mint samples, see Table 1.

Compound	RI_lit_.	RI_exp_.	EOs from Mint Samples—Relative Percentages								
			MpAlL	MpGrL	MpGrF	MpSwL	MpSwF	MpMmL	MpMmF	MsMoL	MsMoF
α-Pinene	936	933	-	0.2	0.1	0.4	0.6	0.5	0.6	0.8	0.9
Sabinene	973	973	0.2 *	0.3	0.1	0.4	0.4	0.5	0.4	0.5	0.5
β-Pinene	978	978	0.9	0.4	0.2	0.6	0.7	0.7	0.7	1.0	0.9
Myrcene	987	990	-	0.9	1.1	0.3	0.3	0.5	0.4	0.7	0.7
3-Octanol	981	1000	0.4	0.1	0.1	0.2	0.2	0.4	0.4	1.4	1.2
α-Terpinene	1013	1015	-	-	-	-	0.1	0.1	-	-	-
*p*-Cymene	1015	1017	-	-	-	0.1	0.1	0.1	0.2	-	-
Limonene	1025	1030	1.8	0.3	0.3	5.8	6.1	0.6	1.9	8.6	10.5
1,8-Cineole	1024	1033	0.8	5.4	2.8	5.7	3.0	4.7	2.8	3.4	2.2
(*E*)-β-Ocimene	1041	1037	-	0.4	0.5	0.9	0.3	0.5	0.1	0.3	0.1
γ-Terpinene	1051	1049	-	-	-	-	-	0.2	-	-	-
(*E*)-Sabinene hydrate	1053	1055	-	-	-	0.9	0.4	1.1	0.7	0.3	0.3
*p*-Mentha-2,4(8)-diene	1077	1077	-	0.6	0.8	-	-	-	-	0.1	-
Terpinolene	1082	1086	-	0.1	0.2	-	-	-	-	-	-
Linalool	1086	1102	1.6	41.2	45.8	0.4	0.2	0.6	0.4	0.4	0.3
1-Oct-3-enyl acetate	1093	1108	-	2.2	1.5	-	-	-	-	-	-
Menthone	1136	1147	-	0.2	0.1	10.8	48.3	48.9	49.3	0.5	0.6
Isomenthone	1146	1167	-	-	-	1.5	5.5	6.5	4.4	-	-
Menthofuran	1150	1168	-	-	-	-	-	-	3.3	-	-
Borneol	1150	1168	1.4	-	-	-	-	-	-	-	-
Neomenthol	1156	1169	-	0.2	0.1	6.8	1.7	2.8	0.9	0.1	0.1
Menthol	1172	1184	-	0.1	0.1	45.2	23.2	22.0	8.6	0.3	0.4
(*Z*)-Dihydrocarvone	1172	1200	-	-	-	-	-	-	-	11.6	2.7
α-Terpineol	1176	1200	-	5.1	5.3	0.2	0.1	-	-	-	-
Dihydrocarveol	1176	1203	-	-	-	-	-	-	-	2.2	0.4
(*E*)-Dihydrocarvone	1177	1207	-	-	-	-	-	-	-	0.5	-
Nerol	1215	1227	-	1.0	1.0	-	-	-	-	-	-
Pulegone	1215	1227	0.8	-	-	-	2.4	1.7	17.5	-	-
Carvone	1215	1247	1.1	0.2	0.2	0.2	0.2	-	-	56.6	66.6
Linalyl acetate	1234	1250	-	24.2	25.7	-	-	-	-	-	-
Geraniol	1235	1253	-	2.5	3.0	-	-	-	-	-	-
Isopiperitenone	1240	1258	2.1	-	-	-	-	-	-	-	-
(*E*)-Carvone epoxide	1243	1266	-	-	-	-	-	-	-	0.3	0.4
Lavandulyl acetate	1275	1269	-	0.2	0.2	-	-	-	-	-	-
Menthyl acetate	1280	1291	-	-	-	11.8	0.5	0.8	0.2	0.2	0.1
Dihydrocarveyl acetate	1314	1326	-	-	-	-	-	-	-	0.7	0.1
(*E*)-Carveyl acetate	1318	1330	-	-	-	-	-	-	-	0.1	-
Piperitenone	1318	1330	26.5	-	-	1.4	1.9	2.0	1.5	-	-
Neryl acetate	1334	1348	0.7	1.4	1.6	-	-	-	-	-	-
Piperitenone oxide	1335	1353	50.0	0.2	0.1	0.1	-	-	-	-	-
Geranyl acetate	1362	1367	-	2.9	3.6	-	-	-	-	-	-
(*Z*)-Jasmone	1371	1375	1.2	0.2	0.1	-	-	-	-	1.2	1.7
Nepetalactone	1379	1382	1.6	-	-	-	-	-	-	-	-
β-Bourbonene	1386	1386	-	-	-	0.3	0.1	0.1	0.1	1.6	1.2
β-Elemene	1389	1390	-	-	-	-	-	-	-	0.1	0.1
β-Gurjunene	1413	1410	-	0.1	-	-	-	-	-	0.1	0.1
(*E*)-β-Caryophyllene	1421	1423	-	0.1	0.1	0.3	0.3	1.1	1.3	1.1	2.2
(*E*)-β-Farnesane	1446	1453	-	0.4	0.3	0.4	0.3	-	-	-	-
α-Humulene	1455	1460	-	-	-	-	-	-	-	0.2	0.4
(*Z*)-Muurola-4(15),5-diene	1462	1466	-	-	-	-	-	-	-	0.3	0.6
Germacrene D	1479	1484	-	1.9	1.0	1.4	1.3	1.3	0.9	0.6	0.9
Bicyclogermacrene	1494	1494	-	-	-	0.6	0.5	0.3	0.1	0.2	0.3
Caryophyllene oxide	1578	1581	-	-	-	-	-	-	-	0.3	0.3
Viridiflorol	1592	1600	-	-	-	-	-	0.2	0.2	-	-
Hinesol	1632	1651	-	0.8	0.6	-	-	-	-	-	-
Isopimara-7,15-diene	2010	2008	1.3	-	-	-	-	-	-	-	-
Total %			92.3	96.7	98.4	96.7	98.7	98.5	98.4	97.1	98.1
Monoterpene hydrocarbons			2.8	3.2	3.3	9.4	9.0	4.8	5.0	12.3	13.9
Oxygenated monoterpenes in total			87.8	85.0	89.7	84.1	87.0	90.0	90.0	78.4	76.0
*Monoterpene ketones*			*81.7*	*0.8*	*0.5*	*14.0*	*58.3*	*59.1*	*72.7*	*70.2*	*72.0*
*Monoterpene alcohols*			*3.0*	*50.1*	*55.3*	*46.7*	*25.6*	*26.5*	*10.6*	*3.3*	*1.5*
*Monoterpene esters*			*0.7*	*28.7*	*31.1*	*11.8*	*0.5*	*0.8*	*0.2*	*1.0*	*0.2*
Sesquiterpenes			-	6.2	3.8	3.0	2.5	3.3	3.0	5.0	7.0
Other compounds			1.7	2.3	1.6	0.2	0.2	0.4	0.4	1.4	1.2
Chemotype			Piperitenone oxide	Linalool		Menthol				Carvone	

* The results are expressed as mean values (*n* = 3).

**Table 3:** Antimicrobial activity of reference compounds and essential oils from leaves and flowers of mint samples. For abbreviations of mint samples, see Table 1.

Mint EOs and Reference Compounds	*Helicobacter pylori* ATCC 43504		Clinical *Helicobacter pylori* Strains			
	mg/L					
	MIC *	MBC	MIC_50_	MIC_90_	MIC Range	MBC Range
MpAlL	15.6	15.6	125	125	62.5–250	15.6–250
MpGrL	31.3	31.3	125	250	62.5–250	31.3–250
MpGrF	31.3	125	250	250	125–500	31.3–500
MpMmL	31.3	31.3	62.5	125	62.5–250	31.3–250
MpMmF	31.3	31.3	250	500	250–500	31.3–1000
MpSwL	15.6	62.5	31.3	125	31.3–125	31.3–125
MpSwF	15.6	62.5	250	500	125–500	62.5–500
MsMoL	31.3	31.3	62.5	62.5	31.3–125	31.3–125
MsMoF	15.6	15.6	62.5	62.5	31.3–125	15.6–125
Menthol	62.5	250	7.8	31.3	7.8–31.3	7.8–250
Menthone	250	250	1000	1000	250–>1000	250–>1000
Linalool	125	250	125	250	62.5–500	62.5–500
1,8-Cineole	125	250	>1000	>1000	>1000	250–>1000
Limonene	62.5	125	500	1000	250–1000	125–1000
(*R*)-(−)-Carvone	62.5	125	31.3	62.5	15.6–125	31.3–125
Dihydrocarvone (*E* + *Z*)	125	125	500	1000	125–1000	125–1000
Metronidazole	31.3	31.3	7.8	31.3	1.95–250	7.8–250

* MIC—minimum inhibitory concentration, MBC—minimum bactericidal concentration, MIC_50_ and MIC_90_—the minimum concentration at which 50% and 90% of the isolates were inhibited, respectively.

**Table 4:** FICI values for selected reference compounds, essential oils, and antibiotics. MpSwL—*M.* × *piperita* ‘Swiss’ leaves; MsMoF—*M. spicata* ‘Moroccan’ flowers.

Analyzed Mint EOs and Reference Compounds	Antibiotics	
	Metronidazole	Clarithromycin
MpSwL	0.28–0.625	0.75–1.00
MsMoF	0.28–0.625	0.62–0.75
Menthol	0.094–0.14	0.51–0.65
(*R*)-(−)-Carvone	0.12–0.31	0.62–0.75

## Data Availability

Not applicable.
